# Synthesis, mol­ecular structure and Hirshfeld surface analysis of (4-meth­oxy­phen­yl)[2-(methyl­sulfan­yl)thio­phen-3-yl]methanone

**DOI:** 10.1107/S2056989018016043

**Published:** 2018-11-16

**Authors:** S. Nagaraju, M. A. Sridhar, C.S. Pradeepa Kumara, M. P. Sadashiva, B. N. Lakshminarayana, N. K. Lokanath

**Affiliations:** a Department of Studies in Physics, Manasagangothri, University of Mysore, Mysore, Karnataka, India; b Department of Studies in Chemistry, Manasagangotri, University of Mysore, Mysuru, Karnataka, India; c Department of Studies in Physics, Adichuchanagiri Institute of Technology, Chikkamagaluru, Karnataka, India; d Department of Studies in Physics, Manasagangotri, University of Mysore, Mysuru, Karnataka, India

**Keywords:** crystal structure, thio­phene, Hirschfeld surface

## Abstract

The title compound, C_13_H_12_O_2_S_2_, crystallizes in the triclinic space group *P* . The mol­ecular structure is substanti­ally twisted, with a dihedral angle of 43.70 (2)° between the 2-(methyl­sulfan­yl)thio­phene and 4-meth­oxy­phenyl rings. In the crystal, mol­ecules are linked through C—H⋯O inter­actions and form a bifurcated layer stacking along the *b-*axis direction and enclosing *R*_2_^2^(10) ring motifs. The phenyl rings are involved in π–π inter­actions with a centroid–centroid separation of 3.760 (2) Å. The Hirshfeld surfaces were studied and the contributions of the various inter­molecular inter­actions were qu­anti­fied.

## Abstract 2

The title mol­ecule is substanti­ally twisted, with a dihedral angle of 43.70 (2)° between the 2-(methyl­sulfan­yl)thio­phene and 4-meth­oxy­phenyl rings. In the crystal, mol­ecules are linked through C—H⋯O inter­actions, forming a bifurcated layer stacking along the *b-*axis direction enclosing 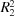
(10) ring motifs.

## Chemical context   

Thio­phenes are five-membered sulfur-containing heterocyclic compounds with important applications in areas such as agrochemistry, pharmaceuticals, mol­ecular electronics, liquid crystalline materials and corrosion inhibition. Thio­phenes are also important building blocks in organic synthesis. Their aromatic character gives enough stabilization to allow the manipulation of various substituents (Mishra *et al.*, 2011 ▸). α-Oxoketene thio­acetals are powerful building blocks for the synthesis of numerous heterocyclic scaffolds, where the carbonyl carbon generally provides hard centers and the carbon-bearing methyl­sulfanyl group acts as a soft electrophilic center (Junjappa *et al.*, 1990 ▸). This synthetic building block was used for the synthesis of (4-meth­oxy­phen­yl) [2-(methyl­sulfan­yl)thio­phen-3-yl]methanone (Pradeepa Kumara *et al.*, 2016 ▸). 
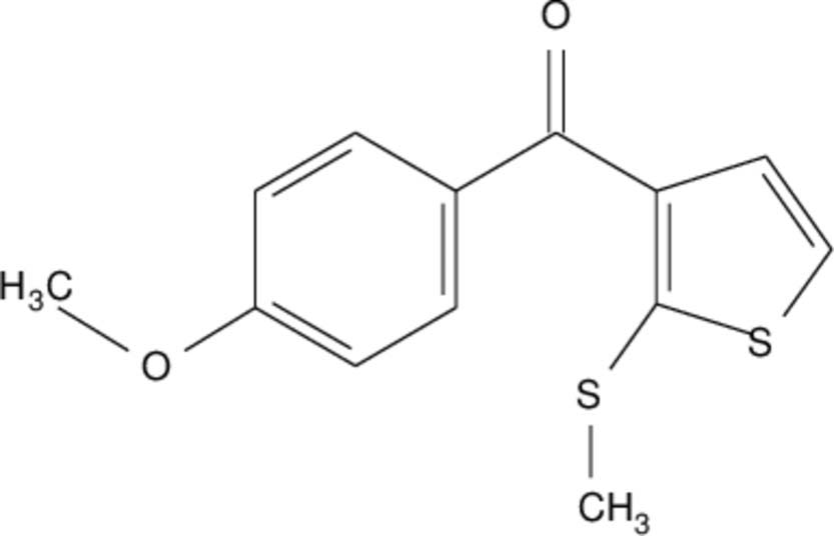


## Structural commentary   

**Figure 1:**
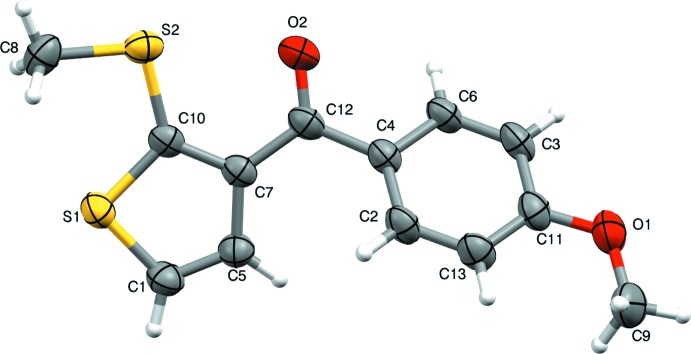
Mol­ecular structure of the title compound, showing the atom-numbering scheme and displacement ellipsoids drawn at the 50% probability level.

In the title compound, the 2-(methyl­sulfan­yl)thio­phene and 4-meth­oxy­phenyl aromatic rings are connected *via* a C(=O)—C methanone bridge (Fig. 1 ▸). The compound is substanti­ally twisted about the methanone bridge as indicated by the dihedral angle of 43.70 (2)° between the thio­phene (S1/C1/C5/C7/C10) and phenyl (C2–C6/C11/C13) rings. The methyl group at S2 is in a +syn-periplanar conformation, as indicated by the C8—S2—C10—S1 torsion angle of 6.09 (16)°. However, in the related compound (4-fluoro­phen­yl)[2-(methyl­sulfan­yl)thio­phen-3-yl]methanone (Nagaraju *et al.*, 2018 ▸), this group is in a -syn-periplanar conformation with a torsion angle of −1.7 (2)°. Atom C12 adopts a nearly trigonal geometry, as indicated by the bond angles C7–C12–O2 = 119.5 (2)°, O2–C12–C4 = 119.2 (2)° and C4—C12—C7 = 121.3 (2)°. The meth­oxy group attached at C11 is in a −anti-periplanar conformation [C3—C11—O1—C9 = −176.9 (2)°]. The bond lengths and angles are normal (Sreenatha *et al.*, 2017 ▸; Rajni Swamy *et al.*, 2014 ▸; Gopinath *et al.*, 2016 ▸).

## Supra­molecular features   

**Figure 2:**
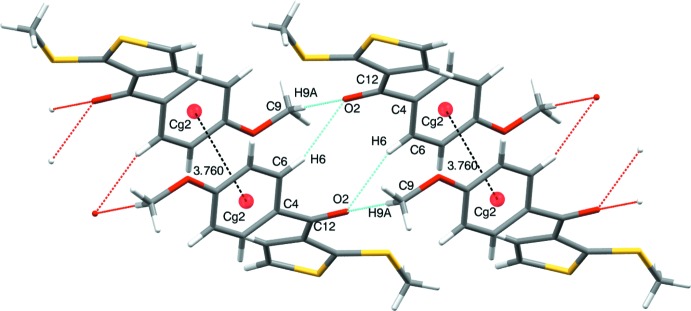
The 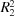
(10) ring motif formed *via* inter­molecular C6—H6⋯O2 hydrogen bonds (Table 1 ▸)*.* The π–π inter­actions are also shown.

**Figure 3:**
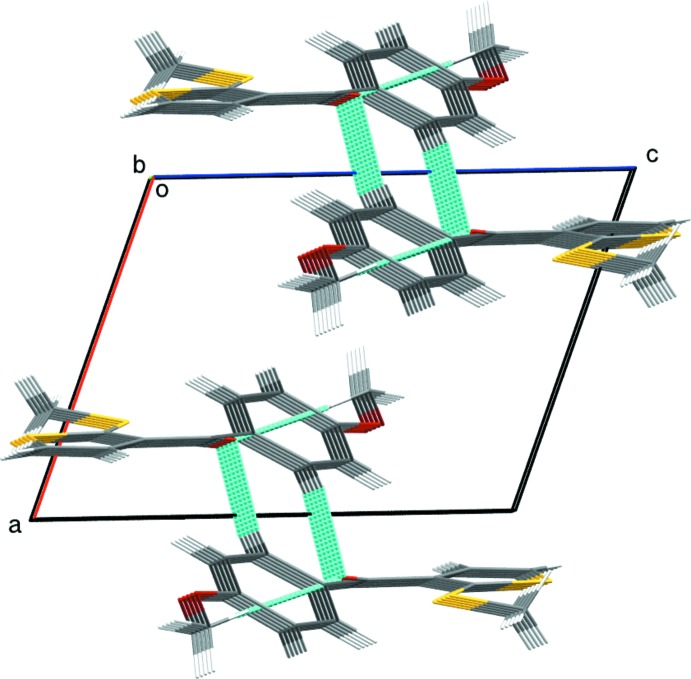
Packing for of the title compound viewed along the *b* axis.

The crystal structure features inter­molecular hydrogen-bonding inter­actions of the type C9—H9*A*⋯O2 (Fig. 2 ▸, Table 1 ▸) and displays a bifurcated layer stacking along the *b-*axis direction through C6—H6⋯O2 inter­actions, which link inversion-related mol­ecules into dimers enclosing an 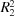
(10) ring motif. π–π stacking inter­actions are also observed between the phenyl rings (centroid *Cg*) of adjacent mol­ecules [*Cg*⋯ *Cg*(2 − *x*, −*y*, 1 − *z*) = 3.760 (2) Å]. The packing of the title compound is illustrated in Fig. 3 ▸.

## Hirshfeld surfaces and 2D fingerprint plots   

**Figure 4:**
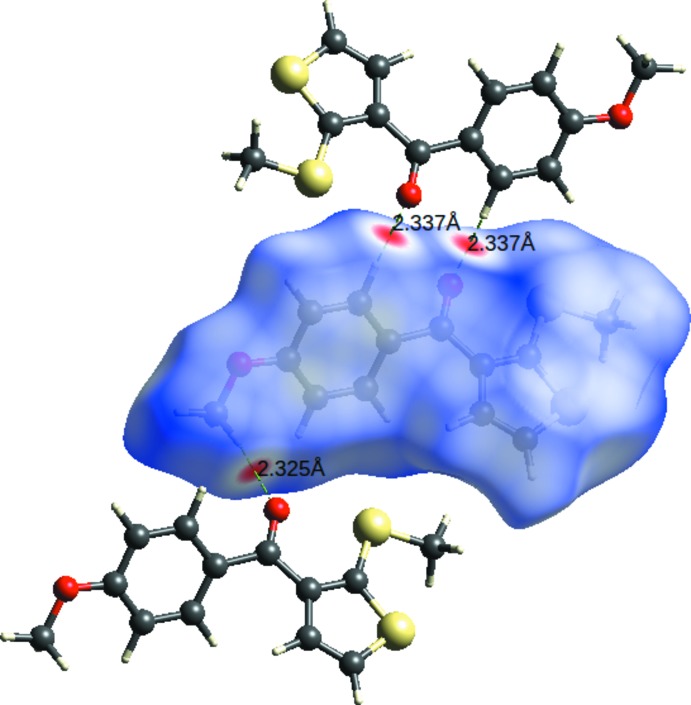
Hirshfeld surface for the title compound mapped over *d*_norm_ in the range −0.2106 to 1.2279 a.u. highlighting the C—H⋯O inter­molecular inter­actions.

**Figure 5:**
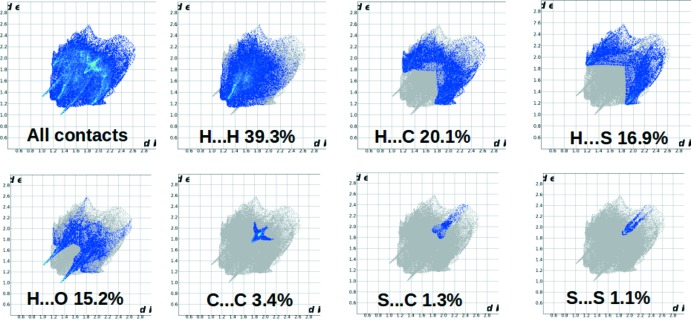
The full two-dimensional fingerprint plots for the title compound, showing (*a*) all inter­actions, and delineated into (*b*) H⋯H, (*c*) H⋯C/C⋯H, (*d*) H⋯S/S⋯H, (*e*) H⋯O/O⋯H, (*f*) C⋯C, (*g*) S⋯C/C⋯S and (*h*) S⋯S inter­actions. The *d*_i_ and *d*_e_ values are the closest inter­nal and external distances (in Å) from given points on the Hirshfeld surface.

**Figure 6:**
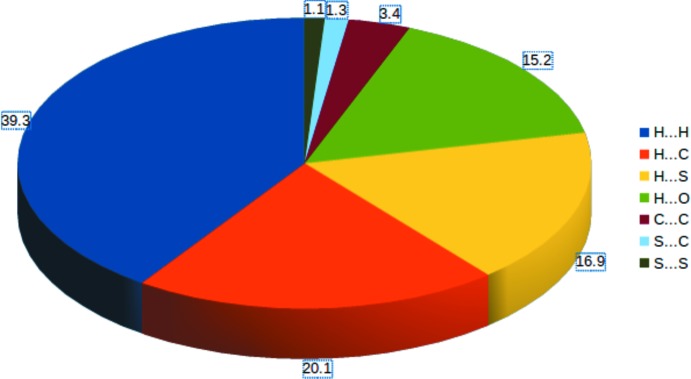
The relative contributions (%) to the Hirshfeld surface for the various contacts.

Hirshfeld surface (HS) analysis and the associated fingerprint plots (FP) (*CrystalExplorer*; Spackman & Jayatilaka, 2009 ▸) are useful tools for visualizing the types of inter­molecular inter­actions present in a crystal structure and qu­antify their percentage contributions to the crystal packing. The 3D HS mapped over *d*_norm_ between −0.2106 a.u (blue) and 1.2279 a.u (red) is shown in Fig. 4 ▸. The area and volume of the HS are 287.29 Å^2^ and 305.24 Å^3^, respectively. The deep-red spots on the *d*_norm_ surface are due to the presence of inter­molecular C—H⋯O inter­actions (Sreenatha *et al.*, 2018 ▸). The 2D FP analysis (Fig. 5 ▸) shows that the H⋯H contacts make the highest contribution (39.3%) followed by the H⋯C/C⋯H contacts (20.1%), which are seen as a pair of blunt spikes in the region 1.2 Å < (*d*_i_ + *d*_e_) < 1.75 Å. The H⋯S/S⋯H contacts make a contribution of 16.9% and appear as butterfly wings in the region 1.2 Å < (*d*_i_ + *d*_e_) < 1.9 Å. The pair of sharp spikes is observed in the region 1.2 Å < (*d*_i_ + *d*_e_) < 1.32 Å is due to the presence of H⋯O/O⋯H contacts (15.6% contribution). The C⋯C contacts (3.4% contribution) are visible as wings in almost the same region, 1.7 Å < (*d*_i_ + *d*_e_) < 1.72 Å. The relative contributions of all the contacts to the Hirshfeld surface are depicted in Fig. 6 ▸.

## Database survey   

A search for thio­phene derivatives was carried out in the Cambridge Structural Database (CSD, Version 5.39, update of February 2018; Groom *et al.*, 2016 ▸). The most relevant compounds are 5-[bis­(4-eth­oxy­phen­yl)amino]­thio­phene-2-carbaldehyde (HOJCIU; Tan *et al.*, 2014 ▸) and 2-[4-(benz­yloxy)phen­yl]-5-(3,4-di­meth­oxy­phen­yl)-3, 4-di­methyl­thio­phene (ACETEI; Shi *et al.*, 2004 ▸), which are both non-planar. In ethyl 4-acetyl-5-anilino-3-methyl­thio­phene-2-carboxyl­ate (AFIGIH; Mabkhot *et al.*, 2013 ▸), the thio­phene and phenyl rings make a dihedral angle of 36.81 (10)°.

## Synthesis and crystallization   

To α-oxoketene di­thio­acetal (0.1 mol) and 1,4-di­thiane-2,5-diol (0.05 mol) in dry ethanol (10 mL), anhydrous potassium carbonate (0.12 mol) was added. The reaction mixture was refluxed on a water bath for 30 minutes (the condenser being protected by a calcium chloride guard tube). After completion of the reaction (monitored by TLC), the catalyst was filtered off and washed with fresh ethanol. The combined ethanol solution was removed on a rotary evaporator to obtain a viscous liquid. The crude product was purified by column chromatography using silica gel with 5% ethyl acetate and petroleum ether to yield the title compound as a yellow solid product, which was recrystallized from di­chloro­methane solution. M.p. 489–493 K. IR (KBr) ν_max_ = 3449, 3079, 2923, 2841, 1772, 1600, 1493, 1253, 1167, 1015, 842, 694, 550 cm^−1^. ^1^H NMR (300 MHz, CDCl_3_): 7.79–7.77 (*m*, 2 H), 7.27–7.25 (*m*, 1H), 7.16–7.14 (*m*, 1H), 6.9–6.93 (*m*, 2H), 3.86 (*s*, 3H), 2.58 (*s*, 3H) ppm. ^13^ C NMR (75 MHz, CDCl_3_): 188.86, 162.73, 151.33, 135.36, 131.60, 131.47, 130.24, 130.59, 122.02, 113.44, 55.37, 18.06. HRMS (ESI): calculated for C_13_H_12_O_2_S_2_ [*M* + H]^+^ 265.0312; found 265.0407.

## Refinement   

Crystal data, data collection and structure refinement details are summarized in Table 2 ▸. All hydrogen atoms were placed at calculated positions and refined using a riding model with C—H = 0.93 Å and *U*_iso_(H) = 1.2*U*_eq_(C) for aromatic ring atoms and with C—H = 0.96 Å with *U*_iso_(H) = 1.5*U*_eq_(C) for methyl groups.

## Supplementary Material

Crystal structure: contains datablock(s) global, I. DOI: 10.1107/S2056989018016043/vm2213sup1.cif

Structure factors: contains datablock(s) I. DOI: 10.1107/S2056989018016043/vm2213Isup2.hkl

CCDC reference: 1871776

Additional supporting information:  crystallographic information; 3D view; checkCIF report

## Figures and Tables

**Table 1:** Hydrogen-bond geometry (Å, °)

*D*—H⋯*A*	*D*—H	H⋯*A*	*D*⋯*A*	*D*—H⋯*A*
C6—H6⋯O2^i^	0.93	2.48	3.374 (4)	161
C9—H9*A*⋯O2^ii^	0.96	2.45	3.400 (4)	172

Symmetry codes: (i) 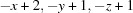; (ii) 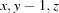.

**Table 2:** Experimental details

Crystal data	
Chemical formula	C_13_H_12_O_2_S_2_
*M*_r_	264.35
Crystal system, space group	Triclinic, *P* 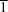
Temperature (K)	293
*a*, *b*, *c* (Å)	7.806 (4), 8.263 (3), 10.414 (6)
α, β, γ (°)	97.260 (11), 109.65 (2), 93.79 (2)
*V* (Å^3^)	623.3 (5)
*Z*	2
Radiation type	Mo *K*α
μ (mm^−1^)	0.41
Crystal size (mm)	0.30 × 0.26 × 0.20

Data collection	
Diffractometer	Bruker APEX
No. of measured, independent and observed [*I* > 2σ(*I*)] reflections	2924, 2165, 1899
*R*_int_	0.109
(sin θ/λ)_max_ (Å^−1^)	0.595

Refinement	
*R*[*F* ^2^ > 2σ(*F* ^2^)], *wR*(*F* ^2^), *S*	0.046, 0.128, 1.09
No. of reflections	2165
No. of parameters	157
H-atom treatment	H-atom parameters constrained
Δρ_max_, Δρ_min_ (e Å^−3^)	0.37, −0.33

Computer programs: *APEX2* (Bruker, 2006 ▸), *SAINT* (Bruker, 2006 ▸), *SHELXS97* (Sheldrick, 2008 ▸), *SHELXL2018* (Sheldrick, 2015 ▸), *PLATON* (Spek, 2009 ▸) and *Mercury* (Macrae *et al.*, 2008 ▸), *PLATON* (Spek, 2009 ▸).

## supplementary crystallographic information

### Crystal data

**Table:**

C_13_H_12_O_2_S_2_	*Z* = 2
*M**_r_* = 264.35	*F*(000) = 276
Triclinic, *P*1	*D*_x_ = 1.409 Mg m^−^^3^
*a* = 7.806 (4) Å	Mo *K*α radiation, λ = 0.71073 Å
*b* = 8.263 (3) Å	Cell parameters from 2924 reflections
*c* = 10.414 (6) Å	θ = 3.5–25.0°
α = 97.260 (11)°	µ = 0.41 mm^−^^1^
β = 109.65 (2)°	*T* = 293 K
γ = 93.79 (2)°	Block, colourless
*V* = 623.3 (5) Å^3^	0.30 × 0.26 × 0.20 mm

### Data collection

**Table:**

Bruker APEX diffractometer	1899 reflections with I > 2σ(I)
Radiation source: graphite	R_int_ = 0.109
Detector resolution: 0.894 pixels mm^-1^	θ_max_ = 25.0°, θ_min_ = 3.5°
SAINT (Bruker, 2006) [not correct; type of scans needed]	h = −9→9
2924 measured reflections	k = −9→9
2165 independent reflections	l = −12→11

### Refinement

**Table:**

Refinement on *F*^2^	Hydrogen site location: inferred from neighbouring sites
Least-squares matrix: full	H-atom parameters constrained
*R*[*F*^2^ > 2σ(*F*^2^)] = 0.046	*w* = 1/[σ^2^(*F*_o_^2^) + (0.072*P*)^2^ + 0.1135*P*] where *P* = (*F*_o_^2^ + 2*F*_c_^2^)/3
*wR*(*F*^2^) = 0.128	(Δ/σ)_max_ < 0.001
*S* = 1.09	Δρ_max_ = 0.37 e Å^−^^3^
2165 reflections	Δρ_min_ = −0.33 e Å^−^^3^
157 parameters	Extinction correction: SHELXL2018 (Sheldrick, 2015), Fc^*^=kFc[1+0.001xFc^2^λ^3^/sin(2θ)]^-1/4^
0 restraints	Extinction coefficient: 0.060 (18)

### Special details

**Table:**

Geometry. All esds (except the esd in the dihedral angle between two l.s. planes) are estimated using the full covariance matrix. The cell esds are taken into account individually in the estimation of esds in distances, angles and torsion angles; correlations between esds in cell parameters are only used when they are defined by crystal symmetry. An approximate (isotropic) treatment of cell esds is used for estimating esds involving l.s. planes.

### Fractional atomic coordinates and isotropic or equivalent isotropic displacement parameters (Å^2^)

**Table:**

	*x*	*y*	*z*	*U*_iso_*/*U*_eq_	
S1	0.77364 (8)	0.32143 (7)	−0.09953 (6)	0.0412 (3)	
S2	0.73606 (8)	0.60555 (7)	0.10012 (6)	0.0422 (3)	
O1	0.7606 (3)	−0.1498 (2)	0.62854 (18)	0.0582 (5)	
O2	0.7950 (3)	0.4642 (2)	0.33504 (18)	0.0601 (5)	
C1	0.8084 (3)	0.1302 (3)	−0.0527 (3)	0.0450 (6)	
H1	0.820956	0.039871	−0.110524	0.054*	
C2	0.6715 (3)	0.0359 (3)	0.3139 (2)	0.0419 (6)	
H2	0.607492	0.011357	0.219380	0.050*	
C3	0.8621 (3)	0.1072 (3)	0.5930 (2)	0.0437 (6)	
H3	0.927331	0.131023	0.687398	0.052*	
C4	0.7766 (3)	0.1880 (3)	0.3679 (2)	0.0361 (5)	
C5	0.8153 (3)	0.1275 (3)	0.0773 (2)	0.0402 (5)	
H5	0.834586	0.034541	0.119774	0.048*	
C6	0.8701 (3)	0.2215 (3)	0.5107 (2)	0.0407 (5)	
H6	0.938609	0.323029	0.550038	0.049*	
C7	0.7900 (3)	0.2813 (3)	0.1446 (2)	0.0352 (5)	
C8	0.6913 (4)	0.6721 (3)	−0.0642 (3)	0.0525 (6)	
H8A	0.594501	0.598579	−0.133310	0.079*	
H8B	0.800026	0.672480	−0.087893	0.079*	
H8C	0.655487	0.781032	−0.059371	0.079*	
C9	0.6614 (5)	−0.3123 (4)	0.5746 (3)	0.0726 (9)	
H9A	0.704580	−0.365458	0.505805	0.109*	
H9B	0.533040	−0.303439	0.533903	0.109*	
H9C	0.681017	−0.375822	0.648323	0.109*	
C10	0.7680 (3)	0.4010 (3)	0.0595 (2)	0.0341 (5)	
C11	0.7571 (3)	−0.0450 (3)	0.5371 (2)	0.0426 (5)	
C12	0.7881 (3)	0.3201 (3)	0.2846 (2)	0.0397 (5)	
C13	0.6597 (3)	−0.0792 (3)	0.3964 (2)	0.0459 (6)	
H13	0.586967	−0.179094	0.358019	0.055*	

### Atomic displacement parameters (Å^2^)

**Table:**

	*U*^11^	*U*^22^	*U*^33^	*U*^12^	*U*^13^	*U*^23^
S1	0.0427 (4)	0.0471 (4)	0.0361 (4)	0.0025 (3)	0.0176 (3)	0.0050 (3)
S2	0.0402 (4)	0.0346 (4)	0.0488 (4)	−0.0026 (2)	0.0148 (3)	0.0010 (2)
O1	0.0729 (13)	0.0652 (12)	0.0423 (10)	0.0013 (10)	0.0272 (9)	0.0124 (8)
O2	0.0870 (15)	0.0460 (10)	0.0429 (10)	0.0040 (9)	0.0214 (9)	−0.0041 (8)
C1	0.0512 (14)	0.0421 (13)	0.0453 (13)	0.0072 (10)	0.0235 (11)	0.0008 (10)
C2	0.0356 (12)	0.0535 (14)	0.0302 (10)	−0.0047 (10)	0.0073 (9)	−0.0002 (9)
C3	0.0400 (12)	0.0615 (15)	0.0258 (10)	0.0005 (11)	0.0106 (9)	−0.0013 (10)
C4	0.0325 (11)	0.0448 (12)	0.0296 (10)	−0.0001 (9)	0.0115 (8)	0.0009 (9)
C5	0.0409 (12)	0.0393 (12)	0.0421 (12)	0.0079 (9)	0.0157 (10)	0.0067 (9)
C6	0.0375 (12)	0.0484 (13)	0.0311 (11)	−0.0036 (10)	0.0110 (9)	−0.0048 (9)
C7	0.0290 (10)	0.0398 (11)	0.0335 (11)	0.0008 (8)	0.0089 (8)	0.0007 (9)
C8	0.0504 (15)	0.0444 (13)	0.0623 (16)	−0.0009 (11)	0.0170 (12)	0.0173 (12)
C9	0.108 (3)	0.0627 (18)	0.0627 (18)	−0.0042 (17)	0.0516 (18)	0.0122 (14)
C10	0.0243 (10)	0.0389 (11)	0.0356 (11)	−0.0033 (8)	0.0096 (8)	−0.0005 (9)
C11	0.0407 (13)	0.0548 (14)	0.0372 (11)	0.0036 (11)	0.0204 (9)	0.0068 (10)
C12	0.0351 (12)	0.0443 (12)	0.0336 (11)	−0.0001 (9)	0.0080 (9)	−0.0026 (9)
C13	0.0409 (13)	0.0528 (14)	0.0387 (12)	−0.0097 (11)	0.0129 (10)	−0.0014 (10)

### Geometric parameters (Å, º)

**Table:**

S1—C10	1.719 (2)	C4—C6	1.400 (3)
S1—C1	1.724 (3)	C4—C12	1.494 (3)
S2—C10	1.744 (2)	C5—C7	1.430 (3)
S2—C8	1.793 (3)	C5—H5	0.9300
O1—C11	1.360 (3)	C6—H6	0.9300
O1—C9	1.448 (4)	C7—C10	1.391 (3)
O2—C12	1.230 (3)	C7—C12	1.458 (3)
C1—C5	1.340 (4)	C8—H8A	0.9600
C1—H1	0.9300	C8—H8B	0.9600
C2—C13	1.379 (3)	C8—H8C	0.9600
C2—C4	1.394 (3)	C9—H9A	0.9600
C2—H2	0.9300	C9—H9B	0.9600
C3—C6	1.365 (3)	C9—H9C	0.9600
C3—C11	1.397 (4)	C11—C13	1.387 (3)
C3—H3	0.9300	C13—H13	0.9300
			
C10—S1—C1	92.19 (11)	S2—C8—H8A	109.5
C10—S2—C8	100.76 (12)	S2—C8—H8B	109.5
C11—O1—C9	117.8 (2)	H8A—C8—H8B	109.5
C5—C1—S1	111.72 (18)	S2—C8—H8C	109.5
C5—C1—H1	124.1	H8A—C8—H8C	109.5
S1—C1—H1	124.1	H8B—C8—H8C	109.5
C13—C2—C4	121.9 (2)	O1—C9—H9A	109.5
C13—C2—H2	119.0	O1—C9—H9B	109.5
C4—C2—H2	119.0	H9A—C9—H9B	109.5
C6—C3—C11	120.7 (2)	O1—C9—H9C	109.5
C6—C3—H3	119.6	H9A—C9—H9C	109.5
C11—C3—H3	119.6	H9B—C9—H9C	109.5
C2—C4—C6	117.6 (2)	C7—C10—S1	110.79 (16)
C2—C4—C12	124.3 (2)	C7—C10—S2	127.08 (17)
C6—C4—C12	118.1 (2)	S1—C10—S2	122.13 (14)
C1—C5—C7	113.6 (2)	O1—C11—C13	125.0 (2)
C1—C5—H5	123.2	O1—C11—C3	115.7 (2)
C7—C5—H5	123.2	C13—C11—C3	119.3 (2)
C3—C6—C4	121.0 (2)	O2—C12—C7	119.5 (2)
C3—C6—H6	119.5	O2—C12—C4	119.2 (2)
C4—C6—H6	119.5	C7—C12—C4	121.33 (19)
C10—C7—C5	111.7 (2)	C2—C13—C11	119.5 (2)
C10—C7—C12	120.6 (2)	C2—C13—H13	120.3
C5—C7—C12	127.7 (2)	C11—C13—H13	120.3
			
C10—S1—C1—C5	0.0 (2)	C8—S2—C10—S1	6.09 (16)
C13—C2—C4—C6	−0.5 (4)	C9—O1—C11—C13	2.7 (4)
C13—C2—C4—C12	−177.3 (2)	C9—O1—C11—C3	−176.9 (2)
S1—C1—C5—C7	0.8 (3)	C6—C3—C11—O1	179.4 (2)
C11—C3—C6—C4	−1.4 (4)	C6—C3—C11—C13	−0.2 (4)
C2—C4—C6—C3	1.7 (3)	C10—C7—C12—O2	−11.1 (3)
C12—C4—C6—C3	178.7 (2)	C5—C7—C12—O2	168.1 (2)
C1—C5—C7—C10	−1.4 (3)	C10—C7—C12—C4	168.49 (19)
C1—C5—C7—C12	179.4 (2)	C5—C7—C12—C4	−12.4 (4)
C5—C7—C10—S1	1.3 (2)	C2—C4—C12—O2	141.9 (3)
C12—C7—C10—S1	−179.42 (16)	C6—C4—C12—O2	−34.8 (3)
C5—C7—C10—S2	−179.26 (16)	C2—C4—C12—C7	−37.6 (3)
C12—C7—C10—S2	0.0 (3)	C6—C4—C12—C7	145.6 (2)
C1—S1—C10—C7	−0.78 (18)	C4—C2—C13—C11	−1.1 (4)
C1—S1—C10—S2	179.77 (14)	O1—C11—C13—C2	−178.2 (2)
C8—S2—C10—C7	−173.3 (2)	C3—C11—C13—C2	1.4 (4)

### Hydrogen-bond geometry (Å, º)

**Table:**

*D*—H···*A*	*D*—H	H···*A*	*D*···*A*	*D*—H···*A*
C6—H6···O2^i^	0.93	2.48	3.374 (4)	161
C9—H9*A*···O2^ii^	0.96	2.45	3.400 (4)	172

Symmetry codes: (i) −*x*+2, −*y*+1, −*z*+1; (ii) *x*, *y*−1, *z*.
